# Occupational asthma due to polyvinyl chloride and methylmethacrylate, “hidden in an adhesive”

**DOI:** 10.1186/2045-7022-3-S1-P32

**Published:** 2013-05-03

**Authors:** Silvia Uriarte Obando, Mar Fernández-Nieto, Joaquín Sastre

**Affiliations:** 1 Fundación Jiménez Díaz, Allergy Department, Spain

## 

Polyvinyl chloride (PVC) is a thermoplastic polymer widely used in industry, reflected in the number of tons consumed annually in the world. We present a 48 year old male patient with no history of atopy, plumber by profession for 30 years, developed progressive dyspnea and dry cough in the last 3 years, triggered in the work environment and persist outside it, related it to manipulate an adhesive called Tangit, whose components are PVC powder and methylmethacrylate. Never presented skin lesions, or used protection. Were performed blood tests, chest radiography, skin tests, spirometry, bronchodilator test and fraction of exhaled nitric oxide, methacholine challenge test was positive (PC 20, 8 mg/dl). After signing informed consent, we performed specific bronchial provocation (SBP) in a 7m^3^ dynamic chamber for 30 minutes of cumulative exposure, simulating working conditions with placebo, the Tangit adhesive provoked a late asthmatic response with a maximal fall in FEV1 of 33% at 7 hours, PVC powder and methylmethacrylate generated dual asthmatic responses, with a maximum drop of 17% at 30 minutes and 17.3% at 7 hours, 22% at 2 minutes and 20% at 9 hours, respectively. The patient during the study was on sick leave from to 24 months. We report a case of occupational asthma by PVC and methylmethacrylate, shown by specific bronchial challenge in a plumber exposed to these agents. There are cases of occupational asthma published by the degradation products of PVC or manipulated workers manufacturing bottle caps1 and packaging. It is the first case of occupational asthma by PVC content in an adhesive. So far been known that adhesives containing acrylates, capable of inducing asthma.

